# Death from the Extraction of a Tooth

**Published:** 1857-07

**Authors:** 


*Death from the Extraction of a Tooth.*—It is stated in a paragraph copied by the Baltimore Sun from the Hartford Times, that Mr. Cooley, a contractor in Colt's pistol factory, died from the effects of having an eye-tooth extracted, three weeks after the operaton had been performed. The jaw and face, however, it seems began to swell soon after the tooth was extracted, causing severe pain. The inflammation and swelling continued until it extended to the throat and head, affecting his nervous system to such a degree, that delirium supervened; those symptoms continued, notwithstanding the most active means were employed to arrest them, until death finally terminated his sufferings. It appears, however, that mortification had previously taken place.

The above is all the information which we have been able to obtain in relation to this most singular and distressing case. It is hoped, however, that the attending physician will furnish a detailed account of it. That such untoward symptoms should have supervened to so simple an operation is certainly very remarkable, and can only be accounted for by supposing the existence of some peculiar irritable condition of the general system of the patient at the time of the extraction of the tooth.

